# Can maxilla and mandible bone quality explain differences in orthodontic mini-implant failures?

**DOI:** 10.1080/26415275.2020.1863155

**Published:** 2021-01-08

**Authors:** Omar Melendres Ugarte, Ivan Onone Gialain, Naor Maldonado de Carvalho, Gisele Lie Fukuoka, Rafael Yague Ballester, Paolo Maria Cattaneo, Marina Guimarães Roscoe, Josete Barbosa Cruz Meira

**Affiliations:** a School of Dentistry, Department of Biomaterials and Oral Biology, University of São Paulo, São Paulo, Brazil; b Department of Dentistry, Section of Orthodontics, Aarhus University, Aarhus, Denmark

**Keywords:** Orthodontic mini-implants, peri-implant bone resorption, finite-element analysis, primary stability

## Abstract

**Purpose:** This study aimed to compare the risk of orthodontic mini-implant (OMI) failure between maxilla and mandible. A critical analysis of finite-element studies was used to explain the contradiction of the greatest clinical success for OMIs placed in the maxilla, despite the higher quality bone of mandible. **Materials and Methods:** Four tridimensional FE models were built, simulating an OMI inserted in a low-dense maxilla, control maxilla, control mandible, and high-dense mandible. A horizontal force was applied to simulate an anterior retraction of 2 N (clinical scenario) and 10 N (overloading condition). The intra-bone OMI displacement and the major principal bone strains were used to evaluate the risk of failure due to insufficient primary stability or peri-implant bone resorption. **Results: **The OMI displacement was far below the 50–100 µm threshold, suggesting that the primary stability would be sufficient in all models. However, the maxilla was more prone to lose its stability due to overload conditions, especially in the low-dense condition, in which major principal bone strains surpassed the pathologic bone resorption threshold of 3000 µstrain. **Conclusions:** The differences in orthodontic mini-implant failures cannot be explained by maxilla and mandible bone quality in finite-element analysis that does not incorporate the residual stress due to OMI insertion.

## Introduction

Mini-implants have become a routine component of the contemporary orthodontists’ anchorage armamentarium [1]. The possibility of preventing unwanted side effects by the skeletal anchorage, the relative ease of placement, the low cost, and the minimal need for patient compliance during active tooth movement have contributed to the increased popularity of this temporary anchorage device (TAD) in orthodontics [1]. The skeletal device stability makes possible the achievement of maximum anchorage to the application of a wide array of orthodontic tooth movements, such as space closure, protraction, retraction, intrusion, extrusion, and to assist in dentofacial orthopedics [2].

An umbrella review recently evaluated the effectiveness of TADs, and a high success rate (≥90%) was reported for most analyzed studies [3]. Failures were associated with infection, OMI fracture, damage to the adjacent structures, and OMI loosening [3]. The OMI loosening can be related to insufficient primary stability or peri-implant bone resorption due to overloading [4]. It seems logical to think that the bone characteristics might strongly influence the degree of OMI primary stability and the risk of peri-implant bone resorption.

Since the beginning of implant therapy, bone quality has gained attention because it was thought to be related directly to the procedure's success. Specifically to OMIs, it is believed that in low-density bones, the OMI installation may fail because it does not achieve sufficient primary stability, which is essential for the mini-implant activation. Still, high primary stability is associated with a zone of dead and dying osteocytes, along with microfractures that lead to a more significant peri-implant bone resorption [5]. Therefore, it is believed that in high-density bones, the primary stability initially achieved can be lost by bone resorption after tissue necrosis due to the surgical steps associated with the OMI installation [6].

The selection of the OMI insertion site is also an important criterion for the clinician [3]. A higher survival rate has been reported for dental implants in the mandible than for those in the maxilla [7,8], and it is probably due to the higher quality of mandible bone. However, orthodontic inter-radicular mini-implants exhibit the opposite outcome. Several studies have reported higher success rates of orthodontic mini-implants in the maxilla, despite the higher quality bone of mandible [9–11]. Undoubtfully, the bone characteristics should be studied comprehensively to understand better their impact on mini-implant therapy [12].

The Finite-Element (FE) Method is a powerful tool to predict and prevent biomechanical failures during treatment planning, including those associated with OMI use [4,13–18]. Still, the accuracy of the method in predicting clinical outcomes depends on the user's ability to insert the correct inputs and select an output coherent with the failure mechanism of interest for the analysis. Although several studies already addressed OMI failures by FEM, significant limitations that impair the predictability of failures are still present. These limitations are related to the nature of bone–OMI contact and the coherency between the proposed failure criteria and the bone-remodeling mechanism induced by the mechanical stimulus.

Concerning the nature of bone–OMI contact, it is essential to highlight that, different from what is desired for a dental implant, OMI osseointegration is not necessary, nor even desirable, because when it occurs, it hinders the device removal and increases the risk of OMI fracture. Regarding the failure criteria, it is already established that the strain is the mechanical stimuli that drive the bone-remodeling process. It was postulated that when strain increases over 3000 µstrains, pathological changes in bone start to present as bone resorption [19]. Frost's theory does not indicate a specific type of strain; therefore, both compression and tension above this threshold of 3000 µε may be related to the peri-implant bone resorption risk.

Nevertheless, due to the chosen failure criterion, most OMI-FE articles limit the analysis to critical tension values [13,14] or critical compression values [4]. Using the maximum principal strain (positive values of ε_1_ corresponds to the maximum tensile strain), the bone resorption risk is restricted to the tension areas. The risk at the compression areas cannot be assessed, although the chances of resorption in these areas are even higher. Therefore, the analysis of the minimum principal strain (negative values of ε_3_ corresponds to the maximum compressive strain) is also crucial. When the major principal strain is used, simultaneous analysis of the compressive and tensile risk areas is performed, which is a considerable benefit.

Therefore, this finite-element study was aimed at assessing the influence of bone density on the risk of OMI loss of primary stability (evaluated by the intra-bone OMI displacement) and peri-implant bone resorption (assessed by the major principal strain in the bone–OMI interface) in the posterior region of the maxilla and the mandible. In addition, a critical analysis of finite-element (FE) studies was used to explain the contradiction of the greatest clinical success for OMIs placed in the maxilla, despite the higher quality bone of mandible.

## Materials and methods

**Figure 1.**
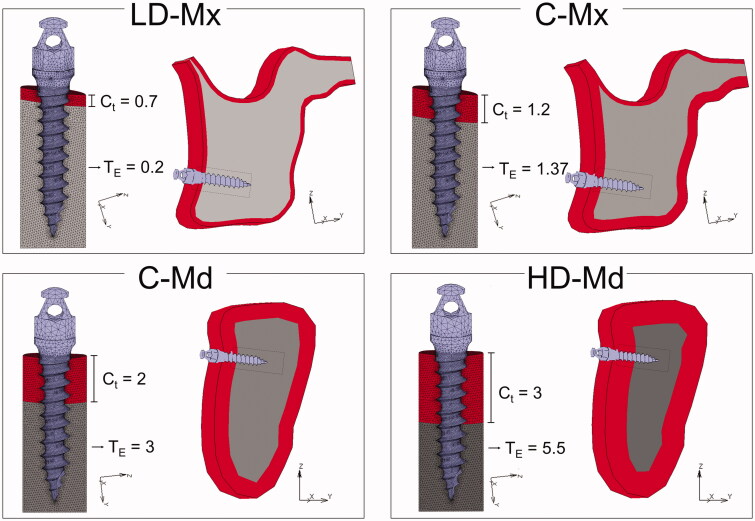
Finite-element models simulating four bone densities conditions: low-dense maxilla (LD-Mx), control maxilla (C-Mx), control mandible (C-Md), and high-dense mandible (HD-Md). Ct: cortical bone thickness (in mm). TE: trabecular bone elastic modulus (in GPa).

Four tridimensional (3 D) finite-element (FE) models of an anatomical maxilla and mandible were built according to four bone densities conditions: low-dense maxilla (LD-Mx), control maxilla (C-Mx), control mandible (C-Md), and high-dense mandible (HD-Md). The bone density differences were simulated by varying the cortical thickness and the trabecular elastic modulus (Figure 1 and Table 1).

**Table 1.** Material properties used in the study.

Materials	Elastic Modulus E (GPa)	Poisson Ratio (γ)
Orthodontic mini-implant (titanium)	113.4	0.3
Cortical bone	13.7	0.3
Trabecular bone		
LD-Mx	0.2	0.3
C-Mx	1.37	
C-Md	3.0	
HD-Md	5.5	

The sagittal external contours of maxilla and mandible bone were based on a CT scan of a 32-year-old man. The use of the patient DICOM file in this research project was approved by the ethics committee of São Paulo University School of Dentistry (registration number 4.251.051). The geometric modeling was performed using Rhino3D (version 5, Robert McNeel & Associates, Seattle, WA), and the mesh generation with Apex (MSC Software, Santa Ana, CA, USA). After a mesh convergence test, the maxilla FE model consisted of 5,92,325 four-node tetrahedral elements and the mandible FE model of 5,76,384 four-node tetrahedral elements. The four 3 D FE models represented a posterior jaw region with a titanium orthodontic mini-implant (9.5 length and 1.4 diameter) inserted in the cortical and trabecular bone of the maxilla (LD-Mx and C-Mx) or mandible (C-Md and HD-Md). The material properties are presented in Table 1. An attrition coefficient of 0.3 was simulated in the interface to simulate a non-osseointegration condition between OMI and bone (condition coherent with the time immediately after the operation).

**Figure 2.**
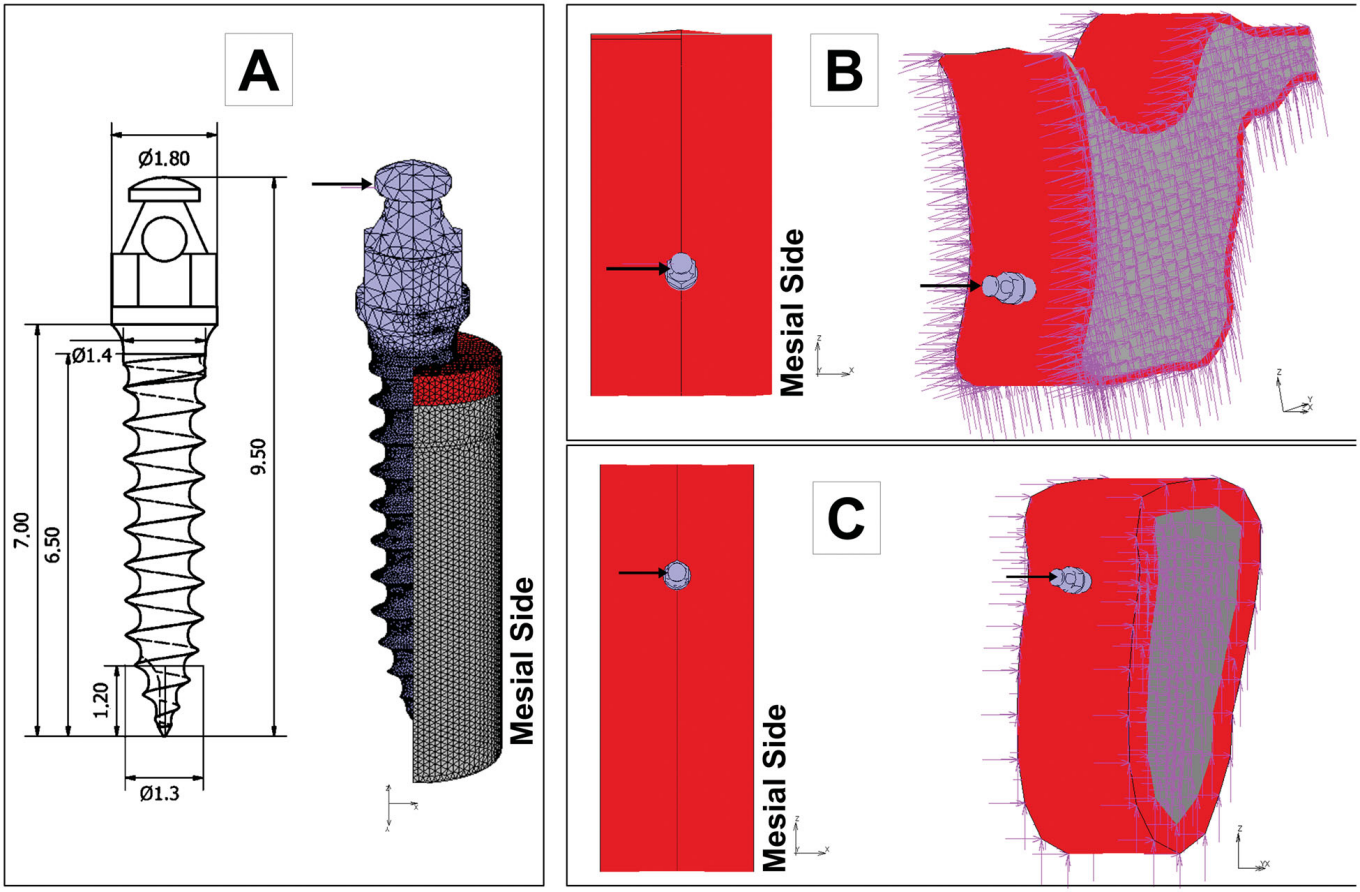
Boundary conditions of the finite element models. A mesial load was applied to the head of the mini-implant, and the displacement of the nodes located at the cutting bone surfaces was restricted (zero degrees of freedom). A: OMI dimensions and loading. B: Maxilla models. C. Mandible models.

An orthodontic force was applied to the head of the OMI (Figure 2) in a horizontal direction to simulate an en-masse retraction of the six anterior teeth. Two load intensities were simulated: 2 N, coherent with the clinical scenario, and 10 N, representing an overloading condition. Constraints (zero degrees of freedom) were assigned to lateral bone cutting faces (Figure 2) that were sufficiently far from the peri-implant bone region.

The numerical analyses were performed with MSC.Marc (MSC Software, Santa Ana, CA, USA). The intra-bone OMI peak of displacement was analyzed to predict the risk of primary stability failure, while compressive and tensile bone major principal strains (ε_M_) were analyzed to predict the risk of peri-implant bone resorption. The ε_M_ value corresponds to the maximum tensile strain or the maximum compressive strain, according to the following criterion:

$$\text{if}\;|\varepsilon_{1}|>|\varepsilon_{3}|\to \varepsilon_{M}=\varepsilon_{1}=\text{ max tensile strain}$$

$$\text{if}\;|\varepsilon_{1}|<|\varepsilon_{3}|\to \varepsilon_{M}=\varepsilon_{3}=\text{ max compressive strain}$$

where $\varepsilon_{1}$ corresponds to the maximum principal strain, and ε_3_ corresponds to the minimum principal strain. It was assumed that either a tensile or a compressive strain above 3000 µstrain would increase peri-implant bone resorption risk.

## Results

### Intra-bone OMI displacement

**Figure 3.**
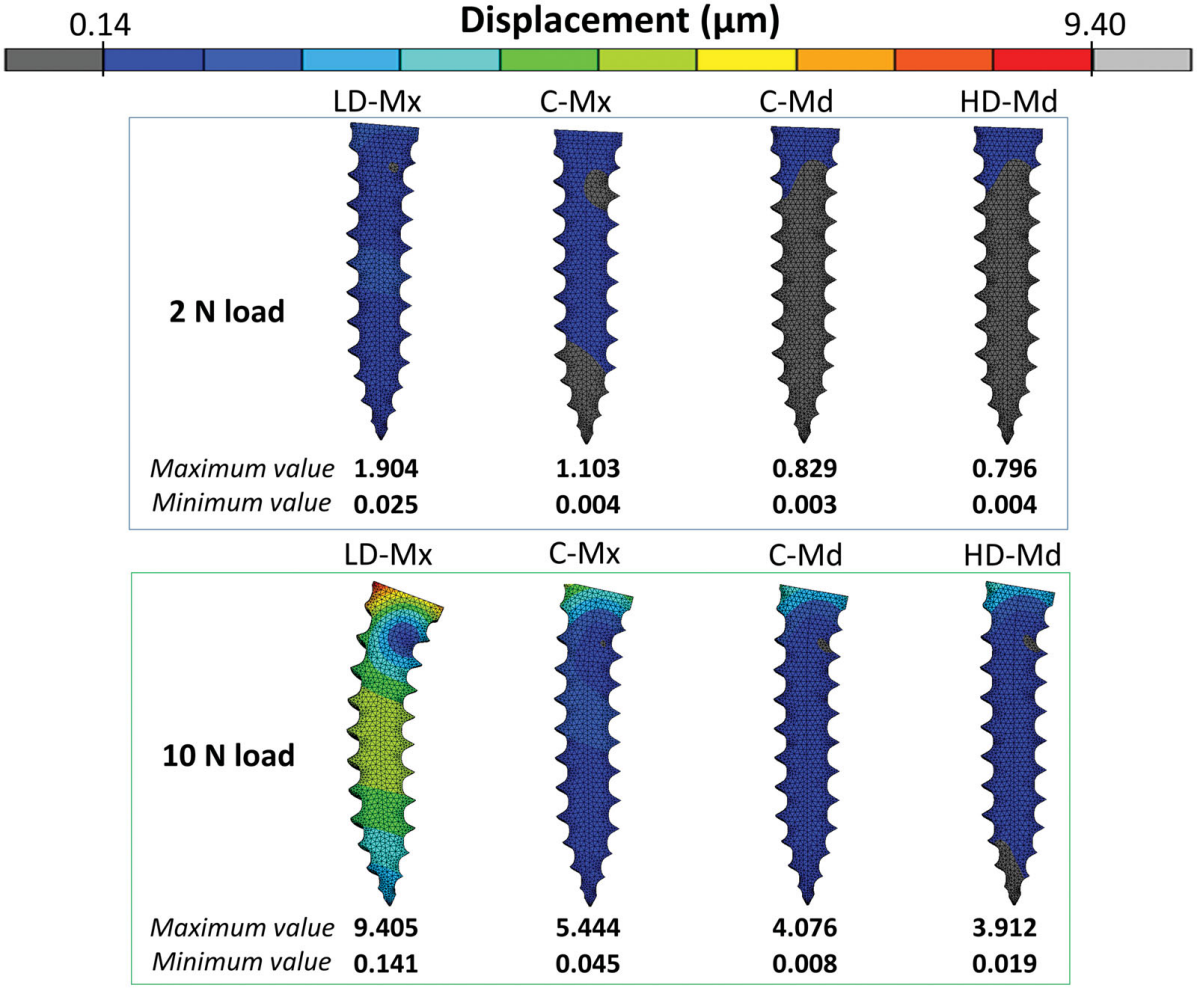
Intra-bone OMI displacement (µm) distribution for the four FE models: low-dense maxilla (LD-Mx), control maxilla (C-Mx), control mandible (C-Md), and high-dense mandible (HD-Md).

The pattern of significant distortion of the OMI body under 2 N and 10 N is presented in Figure 3. The scale adjustment was performed taking the maximum and the minimum displacement values obtained for the LD-Mx model (9.4 µm and 0.1 µm, respectively) as a reference for standardization. Intra-bone OMI displacement increased as the trabecular bone density and the thickness of cortical bone decreased. In the LD-Mx model, the OMI body has deflected more toward the two loadings scenarios simulated. A similar level of primary stability was found between C-Md and HD-Md FE models, which was greater than that of LD-Mx and C-Mx FE models.

### Peri-implant bone major principal strain contours

**Figure 4.**
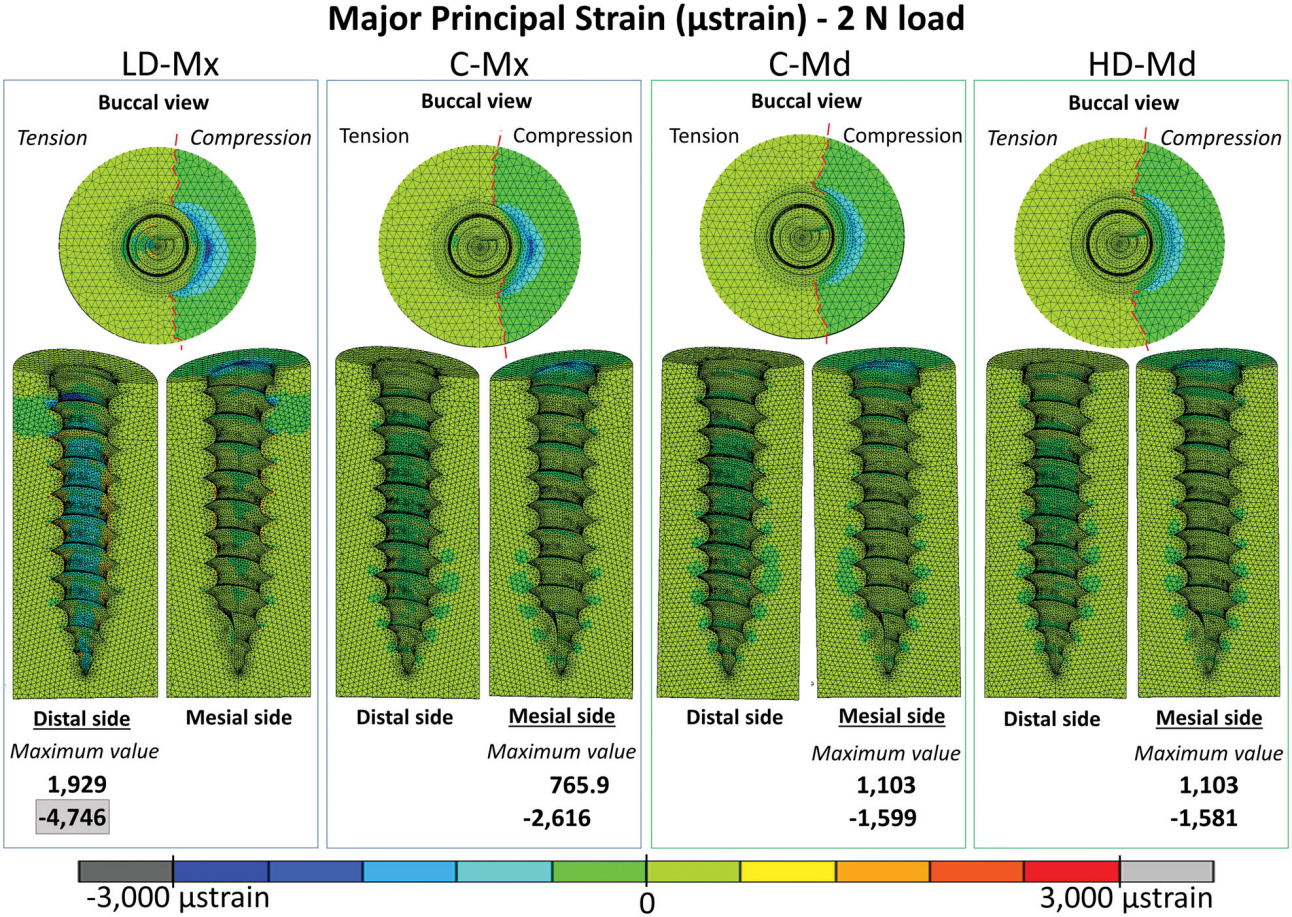
Major strain distribution (µstrain) distribution for the four FE models under 2 N: low-dense maxilla (LD-Mx), control maxilla (C-Mx), control mandible (C-Md), and high-dense mandible (HD-Md).

**Figure 5.**
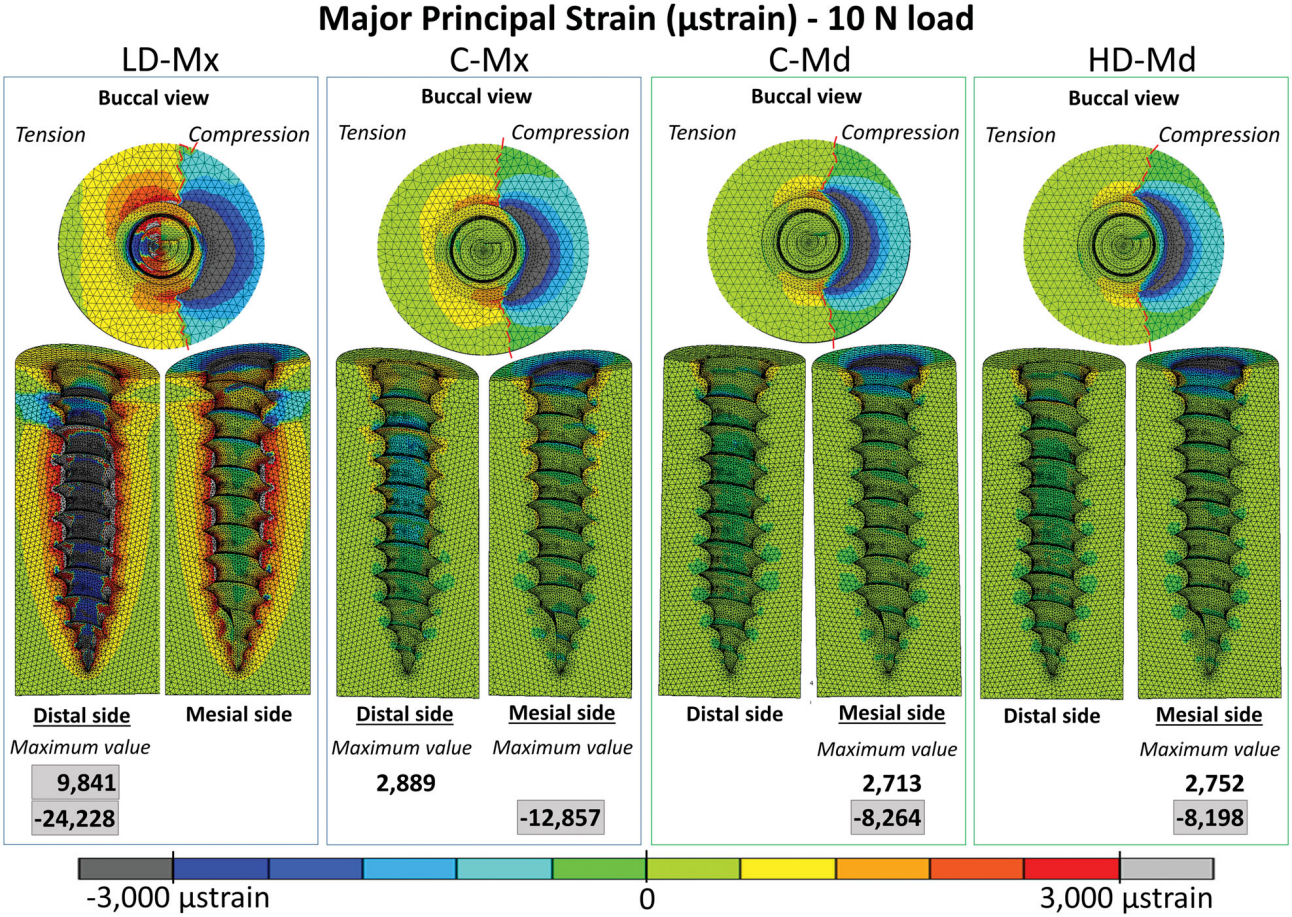
Major principal strain distribution (µstrain) distribution for the four FE models under 10 N: low-dense maxilla (LD-Mx), control maxilla (C-Mx), control mandible (C-Md), and high-dense mandible (HD-Md).

Peri-implant bone major principal strain contours obtained under 2 N and 10 N are presented in Figures 4 and 5, respectively. Compressive and tensile major principal strains decreased as the bone density and the cortical bone thickness increased. In all the FE models, compressive strain peaks were higher than tensile ones. The strain distribution patterns were very similar between C-Md and HD-Md. Regarding the failure criteria, only the LD-Mx model presented strain values that exceeded ±3000 µstrains: 4746 µstrain under 2 N and −24,228 µstrain under 10 N. Still, all FE models exceeded the compressive strain failure criteria with a 10 N load.

## Discussion

Primary stability is defined as the implant stability immediately after OMI insertion, which is mainly dependent on the mechanical interlocking with the cortical bone [10,20]. Clinical studies have shown that the OMI primary stability is influenced by the implant site preparation, the insertion angle, the OMI designs, and the bone quality [20]. Among all these variables, it has been already stated that the most critical factor for the success of OMI stability is the bone quality, being related to physiological and structural aspects, and with the degree of bone tissue mineralization [20]. Some authors assumed that bone quality is equivalent to the bone mineral density, while others have considered that bone quality refers to the cortical thickness [20]. Still, it is a consensus that poor quality and or insufficient quantity of bone can cause a lack of retention of mini-implants.

Maximum insertion torque (MIT) and mobility level have been used as the main parameters to evaluate the primary stability and to assess OMIs success rates in longitudinal clinical studies [10]. In FE studies, the primary stability is commonly evaluated by the OMI displacement [14,17], and a small displacement corresponds to high stability. A direct correlation between FE-displacement and clinical MIT is not feasible. Yet, our FE analysis's general findings corroborate with the clinical [21] and experimental MIT outcomes [22,23]: the displacement decreased as the cortical bone thickness and the trabecular elastic modulus increased.

The observed peak displacement values were compatible with previous FE studies designed with similar loading regime and bone-OMI interface representation [14,16,18]. As far as we know, in the literature, there is no critical displacement value reported able to predict an insufficient OMI primary stability condition. It has been suggested for dental implants a threshold level of tolerated micromotion between 50 and 150 µm, depending on the implant geometry and surface aspects [24]. However, this value serves as a warning to large displacements, which would jeopardize the osseointegration process, favoring the development of fibrous scar tissue around the implant instead of bone apposition, which would compromise the implant stability [24]. It could be expected that the critical displacement threshold for OMIs might be higher compared with the one suggested for dental implants. Two main reasons justify this hypothesis. First, the OMI is usually loaded with a low, static, and unidirectional force, which is less deleterious than the high, dynamic, and multidirectional force of the dental implant [25]. Second, osseointegration is not needed for OMIs. Therefore, a certain degree of mobility can be acceptable [25] as long as it does not compromise the OMI clinical stability and retention.

The intra-bone OMI peak displacement obtained in the present study was far below the 50–100 µm threshold, suggesting that the primary stability would be sufficient even in the overloaded low-dense maxilla scenario. Therefore, clinical failures due to insufficient OMI stability immediately after its insertion could not be confirmed in this study. The low-dense maxilla FE model probably could not represent the critical clinical scenario in which the OMI insertion region presents an ample bone marrow space. Nevertheless, when an excessive load is applied, initially stable mini-implants can become extraordinarily mobile and eventually fail [25]. The stress and strain generated in the peri-implant bone during orthodontic loading stimulate the bone remodeling processes, and the outcomes depend on the stress and strain intensities. According to Frost’s mechanostat theory [19], a 1500–3000 µstrain level would produce a gain in bone mass, since the bone formation process overcome the resorption process. Above 3000 µstrain, the bone would be in a pathologic window, where the resorption process dominates.

Based on the maxilla bone quality (referring to both bone mineral density and cortical thickness), it could be expected that OMIs placed in the mandible would present higher success rates compared with the ones placed in the maxilla. Yet, robust evidence supports the greatest clinical success for OMIs when placed in the maxilla [3,9,10,26]. A recent meta-analysis [10] performed based on 26 clinical studies (5 randomized and 21 non-randomized), and 3934 OMIs reported higher success rates in the maxilla (89%) in comparison to the mandible (82%). These results corroborate what found in a previous systematic review that reported higher success rates for maxilla (87.9% *versus* 80.4% for the mandible) [9], and in a meta-analysis reporting higher failure rates in the mandible (19.3% *versus* 12% for the maxilla) [26].

According to the present results, the maxilla is more prone to lose its stability due to overload conditions, especially in the low-dense condition, in which both tensile and compressive strains surpassed the 3000 µstrain limit. Still, initial high loads should be avoided for all insertion sites, since all FE models exceeded the compressive strain failure criteria under the overloading condition. The compressive strain limit was also reached in the 2 N model of the low-dense maxilla, reinforcing this poor bone quality scenario's vulnerability. It explains why the lack of initial stability is often observed in the case of cortical bone thickness lower than 1 mm [26]. The present results are in agreement with previous FE studies that showed a decrease of the peak stress or strain in peri-implant bone by increasing the cortical bone thickness and/or the trabecular bone elastic modulus [4,15]. Yet, as reported by a recent paper, the effect of cortical thickness on peri-implant stress and strain peaks was not linear, and a thicker cortical bone (C_t_ >2 mm) may not guarantee higher OMIs stability [4]. Neither the increase of cortical thickness from 2 mm to 3 mm nor the denser trabecular bone (Young’s modulus of 3 and 5.5 GPa, respectively) seems to exert a proper advantage to the OMI stability when control (C-Md) and high-dense mandible (HD-Md) models were compared.

The understanding of this apparent contradiction is crucial for clinical decisions in OMI therapies. Although high insertion torque contributes to the implant's immediate stability, it also increases the risk of damaging the interfacial bone during insertion, thus compromising the maintenance of OMI stability [5]. Experimental studies have already shown that OMI insertion stresses and strains are high enough to cause cracks in bone [27]. It is plausible that the negative effect of these interfacial bone stresses and strain rises as the insertion torque and bone stiffness increases [28]. It explains why in Motoyoshi et al. [28] clinical study, the mandible insertion torque was significantly higher in the failure group than in the success group.

The clinical implication of these findings is that for a low-dense maxilla, a higher insertion torque is desirable to guarantee OMI initial stability, while for a dense mandible high insertion torque must be avoided. Therefore, there may be some benefit to predrilling in areas of thick, dense cortical bone, such as the mandible [29], since the insertion torques of self-drilling mini-implants inserted into these regions gradually approach their respective torque values at fracture [21]. Another relevant clinical issue is that the higher stiffness of mandible facilitates the bone overheating during the pre-drilling procedure, mainly when a self-tapping technique is used [30]. On the other hand, when a self-drilling technique is used, the thermal necrosis of bone might be avoided, but a greater microdamage to the cortical bone is observed for both maxilla and mandible [6].

Even though the finite element analysis is a powerful tool to study biomechanical failures, a critical analysis of the loading conditions is crucial for the accuracy of the results. As most published OMI FE studies, the present study was not designed to predict the higher bone resorption vulnerability of the mandible related to the residual strain or the thermal necrosis: our aim was to calculate the strains generated only from the orthodontic forces. As far as we know, only three FE studies simulated the OMI insertion procedure [5,31,32]. All of them simulated a self-tapping technique, and none considered the overheating issue. In Okhyun’s et al. [31] and Yu’s et al. [32] studies, dynamic analyses were performed to simulate the 5 mm advancement of the mini-implant (ø = 1.3 mm) into a 1 mm thick cortical bone disk with a predrilling hole (ø = 0.9 mm). Even though different material models were used to simulate the bone, their main findings were similar: the bone interfacial stresses and strains values were higher than the threshold value triggering pathologic bone resorption. In both articles, an animal model was used to evaluate the reliability of the FE analysis. Also, by a combined animal and FE model study, Cha et al. [5] provided *in vivo* molecular, cellular, and histologic evidence approaching the high OMI insertion torque issues. High bone strains were linked with constriction in blood flow, microfractures in peri-implant bone, and extensive osteocyte death. Although the complex biological responses of the bone due to the OMI insertion were not fully represented in these simulations, it is well defined that the simulation of the strains generated due to implant insertion is crucial for the prediction of the higher risk of failure in high dense mandible by the finite-element method [5].

Other simplifying assumptions used in this study could have affected the accuracy of the FE outcomes. First, the limitation of considering cortical and trabecular bones as a homogeneous material might affect the translation of the FE results to the clinical findings because it has already been shown that homogeneous models presented substantially different strain distributions and magnitudes [14]. Second, although a non-osseointegration condition between OMI and bone was simulated, there was a continuous bone–implant interface in all models, which does not represent the clinical condition, especially in the low-dense maxilla, where large areas of bone marrow are present. Finally, the critical strain of 3000 µ-strain proposed by Frost has been criticized as a valid value of mechanical induced pathologic bone resorption for maxilla and mandible. All these limitations should be addressed in future studies.

## Conclusions

- The intra-bone OMI peak displacement obtained in the present study was far below the 50-100 µm threshold, which suggests that the primary stability would be sufficient even in the overloaded low-dense maxilla scenario.
- The maxilla is more prone to lose its initial stability due to orthodontic overloading, especially in the low-dense condition, in which both tensile and compressive strain surpassed the pathologic bone resorption threshold.
- The differences in orthodontic mini-implant failures cannot be explained by maxilla and mandible bone quality in finite-element analysis that does not incorporate the residual stress due to OMI insertion.
